# Autophagy Induction by Endothelial-Monocyte Activating Polypeptide II Contributes to the Inhibition of Malignant Biological Behaviors by the Combination of EMAP II with Rapamycin in Human Glioblastoma

**DOI:** 10.3389/fnmol.2015.00074

**Published:** 2015-12-01

**Authors:** Jun Ma, Fanjie Meng, Shuai Li, Libo Liu, Lini Zhao, Yunhui Liu, Yi Hu, Zhen Li, Yilong Yao, Zhuo Xi, Hao Teng, Yixue Xue

**Affiliations:** ^1^Department of Neurobiology, College of Basic Medicine, China Medical UniversityShenyang, China; ^2^Institute of Pathology and Pathophysiology, China Medical UniversityShenyang, China; ^3^Department of Neurosurgery, Shengjing Hospital of China Medical UniversityShenyang, China

**Keywords:** EMAP II, autophagy, PI3K/Akt/mTOR, mitophagy, ER stress, rapamycin

## Abstract

This study aims to investigate the effect of endothelial-monocyte activating polypeptide II (EMAP II) on human glioblastoma (GBM) cells and glioblastoma stem cells (GSCs) as well as its possible mechanisms. In this study, EMAP II inhibited the cell viability and decreased the mitochondrial membrane potential in human GBM cells and GSCs, and autophagy inhibitor 3-methyl adenine (3-MA) blocked these effects. Autophagic vacuoles were formed in these cells after EMAP II treatment and this phenomenon was blocked by 3-MA. In addition, the up-regulation of microtubule-associated protein-1 light chain-3 (LC3)-II and the down-regulation of autophagic degraded substrate p62/SQSTM1 caused by EMAP II were observed. Cells treated with EMAP-II inhibited the PI3K/Akt/mTOR signal pathway, and PI3K/Akt agonist insulin-like growth factor-1 (IGF-1) blocked the effect of EMAP II on the expression of LC3-II and p62/SQSTM1. Cells exposed to EMAP-II experienced mitophagy and ER stress. Furthermore, the inhibition of cell proliferation, migration and invasion of GBM cells and GSCs were more remarkable by the combination of EMAP II and rapamycin than either agent alone *in vitro* and *in vivo*. The current study demonstrated that the cytotoxicity of EMAP II in human GBM cells and GSCs was induced by autophagy, accompanied by the inhibition of PI3K/Akt/mTOR signal pathway, mitophagy and ER stress. The combination of EMAP II with rapamycin demonstrated the inhibitory effect on the malignant biological behaviors of human GBM cells and GSCs *in vitro* and *in vivo*.

## Introduction

Glioblastoma (GBM) is the most common and aggressive human brain tumor characterized by local invasion, microvascular proliferation, and therapeutic resistance (Omuro and DeAngelis, 2013). Despite therapeutic advances in the last several decades, the outcome of GBM patients still poor with a median survival of only 12–15 months (Tanaka et al., 2013). Glioblastoma stem cells (GSCs) correspond to a tumor cell subpopulation, display extensive self-renewal, multilineage differentiation, and propagation of tumors (Singh et al., 2004; Bao et al., 2006; Hadjipanayis and Van Meir, 2009; Piccirillo et al., 2009). Considerable evidence have proved that GSCs are responsible for tumor angiogenesis, immune evasion, therapeutic resistance, and tumor recurrence (Bao et al., 2006; Visvader and Lindeman, 2008). Therefore, treatments that improve targeting of GSCs are urgent needed to be provided.

Accumulating evidence showed that different manners of programmed cell death could trigger different biological events. Autophagy, also known as type II programmed cell death, can induce programmed cell death which further propels chemotherapy and/or radiotherapy-mediated cytotoxicity in apoptosis-resistant tumor cells (White, 2012). The role of autophagy in various physiological and pathological processes is currently being investigated. Drug-induced autophagy has been corroborated to have anti-inflammatory and anti-tumor effects, and it has been confirmed that the autophagic response could strengthen the anti-tumor effects of drugs (Chen and Karantza-Wadsworth, 2009; Al-Ejeh et al., 2010). Therapeutic studies have shown that glioma cells could undergo autophagy by drugs, which triggers autophagic cell death. Clinical trials proved that temozolomide (TMZ), which achieved better curative effects in the patients with apoptosis-resistant GBM (Kanzawa et al., 2004), could induce autophagy in GBM cells as well (Minniti et al., 2008). Valproic acid (VPA) was also confirmed to be able to cause cell death of glioma cell lines by inducing autophagy (Fu et al., 2010). Recent study also showed that cannabidiol inhibits GSCs proliferation by inducing autophagy in a transient receptor potential vanilloid-2(TRPV2)-dependent manner (Nabissi et al., 2015). Taken together, inducing autophagic death might be one of the main mechanisms of anti-neoplastic drugs in human GBM cells and GSCs.

Endothelial-monocyte-activating polypeptide II (EMAP II) is a protein extrated from methylcholantherene A (Meth A) transformed fibrosarcoma (Kao et al., 1994). Low-dose EMAP II has been confirmed to increase the blood-tumor barrier (BTB) permeability by opening tight junction (TJ) via Rho A, PKC or other signal pathways (Li et al., 2012, 2015; Xie et al., 2012). Furthermore, EMAP II has been confirmed as a component of the immunosuppressive pathway in several solid tumors (Murray et al., 2004a,b; Faisal et al., 2007). EMAP II could suppress the growth of primary and metastatic tumors and induce apoptosis in growing endothelial cells (Schwarz et al., 2010a). Elevated EMAP II expression was associated with lymphocyte apoptosis and lack of metastases in colon cancer patients (Stathaki et al., 2014). The *in vivo* study reported that EMAP II attenuated the primary tumor growth of rat C6 glioma cells (Schwarz and Schwarz, 2004). In pancreatic cancer, EMAP II alone or in combination with bortezomib have anti-proliferative and pro-apoptotic effects, and significantly reduced B-cell lymphoma-2 (Bcl-2) expression (Awasthi et al., 2010). Beclin-1 was found to induce autophagy, and Bcl-2 could inhibit its function by binding its BH_3_ domain (Guertin and Sabatini, 2005), suggesting that EMAP II might induce autophagy. Therefore, whether EMAP-II induces the autophagy of human GBM cells and GSCs needs to be explored urgently.

In the present study, we investigated whether EMAP II induce cell autophagy in human GBM cells and GSCs, and its potential mechanisms. Further, we examined the effect of the combination of EMAP II with rapamycin on biological behaviors of human GBM cells and GSCs *in vitro* and *in vivo*.

## Materials and Methods

### Reagents

Dulbecco’s modified eagle’s medium (DMEM), fetal bovine serum (FBS), DMEM/F-12 medium and basic fibroblast growth factor (bFGF), epidermal growth factor (EGF), 2% B27 and MitoTracker Deep Red FM were purchased from Life Technologies Corporation (Carlsbad, CA, USA). EMAP II, rapamycin, dimethyl sulfoxide (DMSO), 3-methyl adenine (3-MA), Z-VAD-fmk (Z-VAD), tauroursodeoxycholate (TUDC), and laminin were purchased from Sigma–Aldrich (St. Louis, MO, USA). Cell counting kit-8 (CCK8), DAPI, Alexa Fluor 488, Alexa Fluor 555, and BCA protein assay kit were purchased from Beyotime Institute of Biotechnology (Jiangsu, China). 5,5,6,6′-tetrachloro-1,1′,3,3′-tetraethyl-benzimidazol-carbo-cyanine iodide (JC-1) apoptosis detection kits were purchased from Becton Dickinson Bioscience (San Jose, CA, USA). IGF-1 was purchased from PeproTech (St.Louis, MO, USA). Primary antibodies against microtubule-associated protein 1 light chain 3 (LC3, ab63817) and p62/SQSMT1 (ab56416) were purchased from Abcam (Cambridge, MA, USA). Primary antibodies against p-PI3K (#4228), PI3K (#4257), p-Akt (Ser473) (#9271), Akt (#9272), p-mTOR (Ser 2448) (#2971), mTOR (#2972), GRP78 (#3183), p-eIF2α (#3398), eIF2α (#5324), and CHOP (#5554) antibodies were from Cell Signaling Technology (Beverly, MA, USA). Anti-GAPDH (sc-365062) and the secondary antibodies conjugated with horseradish peroxidase were purchased from Santa Cruz Biotechnology (Santa Cruz, CA, USA). Primary antibodies against TOMM20 (612278), TIMM23 (611222), and Matrigel were purchased from BD Biosciences (Franklin Lakes, NJ, USA).

### Cell Culture and Treatment Conditions

Human GBM cell lines (U87 and U118) were obtained from Shanghai Institutes for Biological Sciences Cell Resource Center and were cultured in DMEM with 10% FBS in a humidified atmosphere of 5% CO_2_ at 37°C. GSCs were isolated from fresh surgical specimens of human GBM tissues following the procedures as described previously (Singh et al., 2003; Yao et al., 2015). GSCs were maintained in DMEM/F-12 medium supplemented with bFGF (20 ng/ml), EGF (20 ng/ml) and 2% B27. The five independent GSCs were isolated from 5 GBM patients. All the patients provided written informed consent, and the study was approved by the Ethics Committee of Shengjing Hospital of China Medical University. Only early passage cell line were used for the study. Information of patient tissues was listed in Supplementary Table S1.

For the experiments, cells were treated with EMAP II at different concentrations (0.005 nM, 0.05 nM, 0.5 nM, and 5 nM, diluted with saline solution) for 0.5, 1, 2, 3, and 6 h, and then the medium was replaced with fresh medium for 24 h. However, cells in control group were maintained in medium containing the same concentration of saline. According to the related results, 0.05 nM and 0.5 h was considered as the optimum dosage and time, respectively. Furthermore, cells were pretreated with 2 mM 3-MA, 100 μM Z-VAD, 2 mM 3-MA+100 μM Z-VAD, 10 nM IGF-1, 500 μM TUDC, and 50 nM rapamycin in different experiments in this study.

### Cell Viability Assay

Cell viability was measured using CCK8 according to the manufacturer’s instructions. The principle of the cell viability assay is that mitochondrial activity is constant for most viable cells and thereby an increase or decrease in the number of viable cells is linearly related to mitochondrial activity. CCK8 is based on the WST-8, which is reduced by dehydrogenases in the cells giving an orange colored formazan. The mitochondrial activity of the cells is reflected by the formazan. Thus, any increase or decrease in viable cell number can be detected by measuring formazan concentration. Briefly, cells were seeded in 96-well plate with 200 μl medium, incubated for 24 h under standard conditions and treated with reagents for indicated time. At the end of the time point, 20 μl of CCK8 was added to each well and incubated for additional 2 h, and the absorbance was finally measured at the wavelength of 450 nm.

### JC-1 Assay for Flow Cytometry

Cells were seeded in 6-well plate with 2 ml medium, incubated for 24 h and then treated with reagents for indicated time. After incubated with 10 μg/ml JC-1 at 37°C for 30 min, cells were washed once with PBS and resuspended with 500 μl PBS. The samples were immediately measured by flow cytometer. In living cells, JC-1 accumulates in mitochondria forming red fluorescent aggregates, JC-1 exists mainly in green fluorescent monomeric form once mitochondria membrane potential is depolarized. The results were analyzed by cell quest software. JC-1 green and red fluorescence was recorded on FL1 and FL2 channels, respectively. The dots distributed in the upper right quadrant (UR) and lower right quadrant (LR).

### Transmission Electron Microscopy (TEM) Analysis

Cells were fixed with 2.5% glutaraldehyde at 4°C for 2 h and subsequently fixed with 1% OsO_4_-0.15 M Na cacodylate/HCl (pH 7.4) for 30 min. Then cells were dehydrated in graded ethanol and polymerized at 60°C for 48 h. Samples were prepared and analyzed with a JEM 1230 transmission electron microscope (JEM-1200EX; *n* = 5, each) at 60 kV. Micrographs were taken at ×5,000 or at ×10,000.

### Immunofluorescence Assay

The U87 and U118 cells were seeded on chamber slides (or GSCs were seeded on chamber slides pre-coated with fresh laminin for adherent culture) for 24 h, then treated with 0.05 nM EMAP II for 0.5 h or 0.05 nM EMAP II for indicated time. Cells were stained with LysoTracker Red at a final concentration of 50 nM, or MitoTracker Deep Red FM at a final concentration of 100 nM. Cells were fixed in 4% paraformaldehyde for 20 min, and then incubated with 5% BSA for 2 h at room temperature. Primary antibody staining was performed for LC3 (or LC3 and p62/SQSMT1). After that, cells were washed and incubated with a secondary antibody conjugated to Alexa Fluor 488 and Alexa Fluor 555. Nuclei were stained with 0.5 μg/ml DAPI. The cells were visualized using immunofluorescence microscopy (Olympus, Tokyo, Japan).

### Western Blot Assay

Cells treated with reagents for indicated time were lysed in RIPA buffer supplemented with a proteinase inhibitor (10 mg/ml aprotinin, 10 mg/ml phenyl-methylsulfonyl chloride (PMSF) and 50 mM sodium orthovanadate). Cell extracts were quantified using the BCA protein assay kit and equal amounts of proteins were separated by SDS-PAGE. Gels were then transferred to PVDF membranes, blocked with 5% non-fat dry milk and incubated with the primary antibody solution. Alternatively, primary phospho-antibodies were diluted in 5% BSA in TBST overnight at 4°C. The membrane was washed with TBST and incubated with an HRP-conjugated secondary antibody solution. After subsequent washes, immunoblots were visualized by enhanced chemiluminescence (ECL kit, Santa Cruz Biotechnology). Autoradio-graphic images were scanned and integrated density value (IDV) of protein bands were quantified by ChemImager 5500 software.

### Scratch Wound Healing Assay

Scratch wound healing assay was adapted to evaluate the migration ability of U87 and U118 cells. Briefly, cells were seeded on 6-well plates at the density of 1 × 10^4^ per well until they reached 80% confluence. Scratching wounds were created in the monolayer of confluent cells with a pipette tip. The width of wounds was assessed to be the same at the beginning of the experiments. The wells were rinsed with PBS three times to remove floating cells and debris. Cells were treated with EMAP II, rapamycin or the combination of EMAP II and rapamycin at indicated time. Wound healing was measured and recorded photographically.

### Transwell Assays

The migration and invasion abilities of U87, U118, and GSCs were detected using 24-well transwell chambers with 8 μm pore size (Corning Costar). Cells were resuspended in medium and seeded on the upper chambers (without Matrigel for cell migration assay) or seeded on the upper chambers pre-coated with Matrigel (for cell invasion assay). After incubated for 24 h, the inserts were taken out and cells remained on the upper surface of the filters were removed carefully with a cotton wool swab. The migrated and invaded cells were washed with PBS and fixed with methanol and glacial acetic acid (mixed at 3:1) for 30 min at room temperature and stained using Giemsa stain for 15 min. The average number of cells was counted in six random fields.

### *In Vivo* Xenograft Study

Four-week-old BALB/C athymic nude mice were supplied by Cancer Institute of the Chinese Academy of Medical Science. All experiments were carried out under the approval of the Administrative Panel on Laboratory Animal Care of Shengjing Hospital. All animals had access to food and water ad libitum. For subcutaneous implantation, a total of 3 × 10^5^ cells suspended in 100 μl medium was injected into the right flank regions of nude mice. Tumor volume was calculated according to the formula: volume (mm^3^) = (a × b^2^)/2 (where a is the largest superficial diameter and b is the smallest superficial diameter). When the tumors grew to about 80 mm^3^, the tumor-bearing mice were randomly assigned to the control group (treated with 0.05 nM saline), EMAP-II treatment group (treated with 80 ng/kg EMAP-II), rapamycin treatment group (treated with 50 nM rapamycin), and the combination of EMAP-II and rapamycin group (treated with 50 nM rapamycin and 80 ng/kg EMAP-II). The tumor-bearing mice were treated as described above every 12 h for 18 weeks. Tumor volume was measured every other day for 18 weeks.

### Statistical Analysis

All results were expressed as mean ± SD for each group and analyzed by use of SPSS 18.0 software. One-way ANOVA and post hoc comparisons (Bonferroni test) were utilized to determine the significant differences among multiple groups. *P* < 0.05 was considered statistically significant.

## Results

### Cytotoxicity of EMAP II in Human GBM Cells and GSCs

**FIGURE 1:**
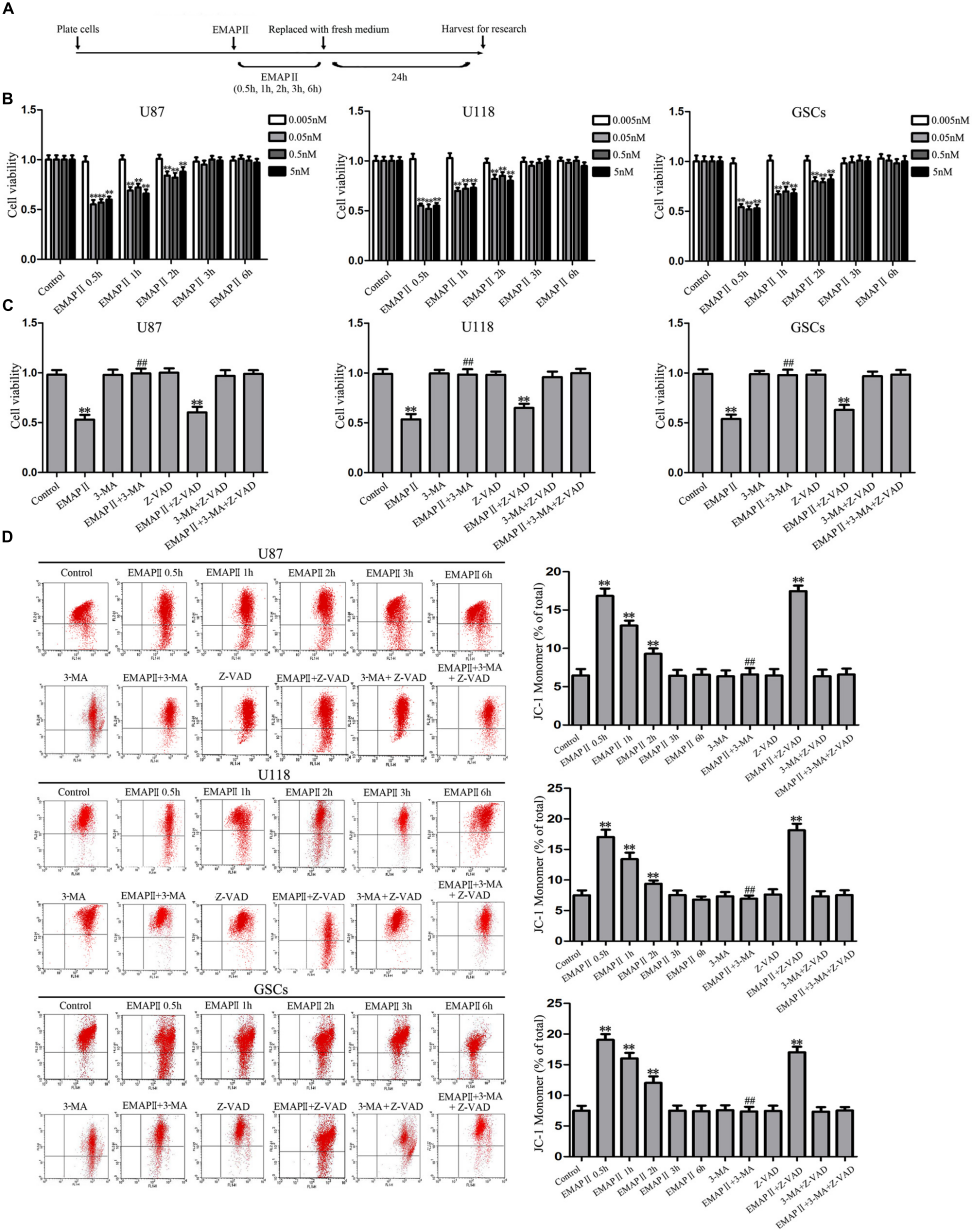
**Effect of endothelial-monocyte activating polypeptide II (EMAP II) on human GBM cells and GSCs. (A)** Timeline of the research with EMAP II. **(B)** Effect of EMAP II on the cell viability of U87, U118, and GSCs after treatment with 0.005 nM, 0.05 nM, 0.5 nM, and 5 nM EMAP II for 0.5, 1, 2, 3, and 6 h. **(C)** Effects of EMAP II, 3-MA and Z-VAD on the cell viability of U87, U118 and GSCs after treatment with 0.05 nM EMAP-II for 0.5 h. **(D)** Effects of EMAP II, 3-MA, and Z-VAD on MMP of U87, U118, and GSCs by JC-1 staining for flow cytometry. Values represented the means ± SD (*n* = 5, each). ^∗∗^*P* < 0.01 vs. control group, ^##^*P* < 0.01 vs. EMAP II group.

The effects of EMAP II on the cell viability of human GBM cells and GSCs were evaluated at indicated time and concentration (the time line of experiments was shown in **Figure 1A**). As shown in **Figure 1B**, the cell viability of U87, U118, and GSCs were inhibited by EMAP II in time- and dose-dependent patterns. Compared with the control group, the cell viability was significantly decreased at 0.5 h with the treatments of 0.05, 0.5, and 5 nM EMAP II (*P* < 0.01). The cell viability of U87, U118, and GSCs were inhibited at 0.5, 1, and 2 h in groups of 0.05, 0.5, and 5 nM, respectively (*P* < 0.01), whereas there was no obvious difference among three groups (*P* > 0.05). Thus, 0.05 nM at 0.5 h were selected as the optimum concentration and time point in the subsequent experiments, respectively. We further investigated whether the inhibitory effect of EMAP II on cell viability was associated with the induced autophagy and apoptosis. Cells were pretreated with autophagy inhibitor 3-MA or caspase inhibitor Z-VAD alone or in combination. As shown in **Figure 1C**, the cell viability was inhibited in EMAP II and EMAP II+Z-VAD groups compared with control group (*P* < 0.01), and there was no significant difference between these groups (*P* > 0.05). In addition, the cell viability was increased in EMAPII+3-MA group compared with EMAP II group (*P* < 0.01), suggesting that 3-MA blocked the inhibitory effect of EMAP II on the cell viability.

JC-1 staining was used to further detect the effect of EMAP II on mitochondrial membrane potential (MMP) in human GBM cells and GSCs. As shown in **Figure 1D**, results showed that the ratio of JC-1 monomer increased significantly and reached a peak at 0.5 h (*P* < 0.01). Then it decreased, and finally returned to the level of control group. Since the increase of JC-1 monomer ratio is an indicator of MMP, the above result demonstrated that EMAP II decreased the MMP of U87, U118, and GSCs in a time-dependent manner (*P* < 0.01). In addition, the ratio of JC-1 monomer in EMAP II +3-MA group was decreased compared with that in EMAP II group, suggesting that 3-MA blocked the EMAP II-induced decrease in MMP. The above results suggested that inhibitory effects of EMAP II on the cell viability and MMP might be associated with cell autophagy in human GBM cells and GSCs.

### EMAP II Induced Cell Autophagy in Human GBM Cells and GSCs

**FIGURE 2:**
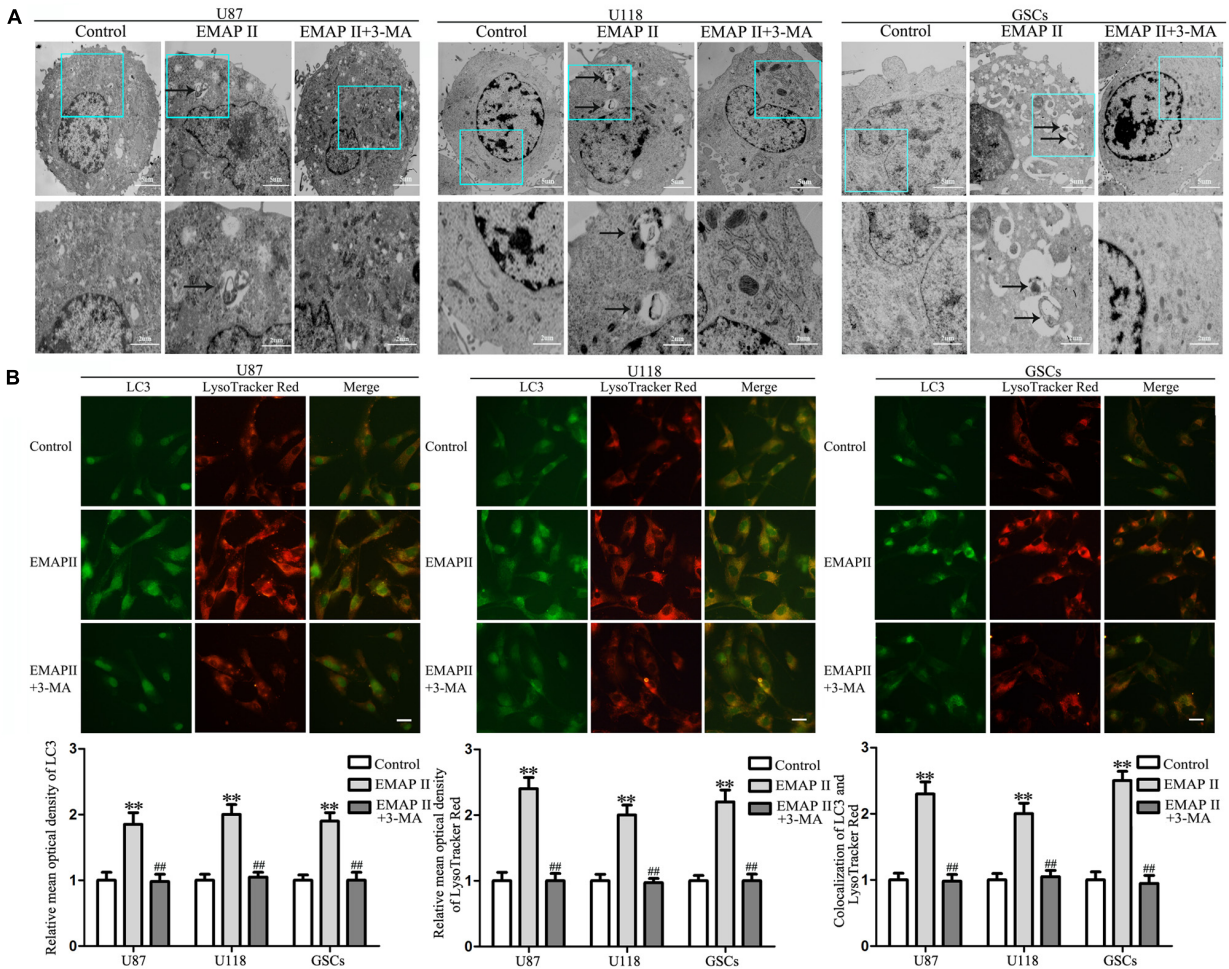
**Effect of EMAP II and 3-MA on the ultrastructural change in human GBM cells and GSCs. (A)** Effect of EMAP II and 3-MA on the ultrastructural change in U87, U118, and GSCs. Arrows show autophagic vacuoles. **(B)** The colocalization of LC3 and LysoTracker Red in U87, U118, and GSCs. Relative mean optical density of LC3 and LysoTracker Red, and the co-localization of LC3 and LysoTracker Red quantified. Pictures are respective magnification (*n* = 4, each). Scale bar = 20 μm. ^∗∗^*P* < 0.01 vs. control group, ^##^*P* < 0.01 vs. EMAP II group.

Due to the fact that the cell viability and MMP were not changed in cells treated with 3-MA alone, the following experiments were performed without cells treated with 3-MA alone. TEM was used to observe the effects of EMAP II on the ultrastructural changes in human GBM cells and GSCs. As shown in **Figure 2A**, the autophagic vacuoles were positive in U87 cells in the 0.05 nM EMAP II for 0.5 h group and negative in the control group and the EMAP II+3-MA group. The same results were also observed in U118 cells and GSCs. As shown in **Figure 2B**, cells were stained with anti-LC3 and LysoTracker Red by immunofluorescence. EMAP II significantly increased the punctate distribution and density of LC3 as well as autophagic vacuoles numbers in U87, U118, and GSCs (*P* < 0.01). Co-treatment with 3-MA reduced the punctate distribution and density of LC3 as well as autophagic vacuoles numbers (*P* < 0.01). In addition, we found that there was a significant overlap between LC3 and lysosomal signals in EMAP II treated cells (*P* < 0.01), suggesting that autophagosome–lysosome fusion was not inhibited by EMAP II. These results suggested that EMAP II could induce autophagic vacuoles formation in human GBM cells and GSCs, and 3-MA could block this effect.

### EMAP II Regulated the Expression of LC3 and p62/SQSTM1 in Human GBM Cells and GSCs

**FIGURE 3:**
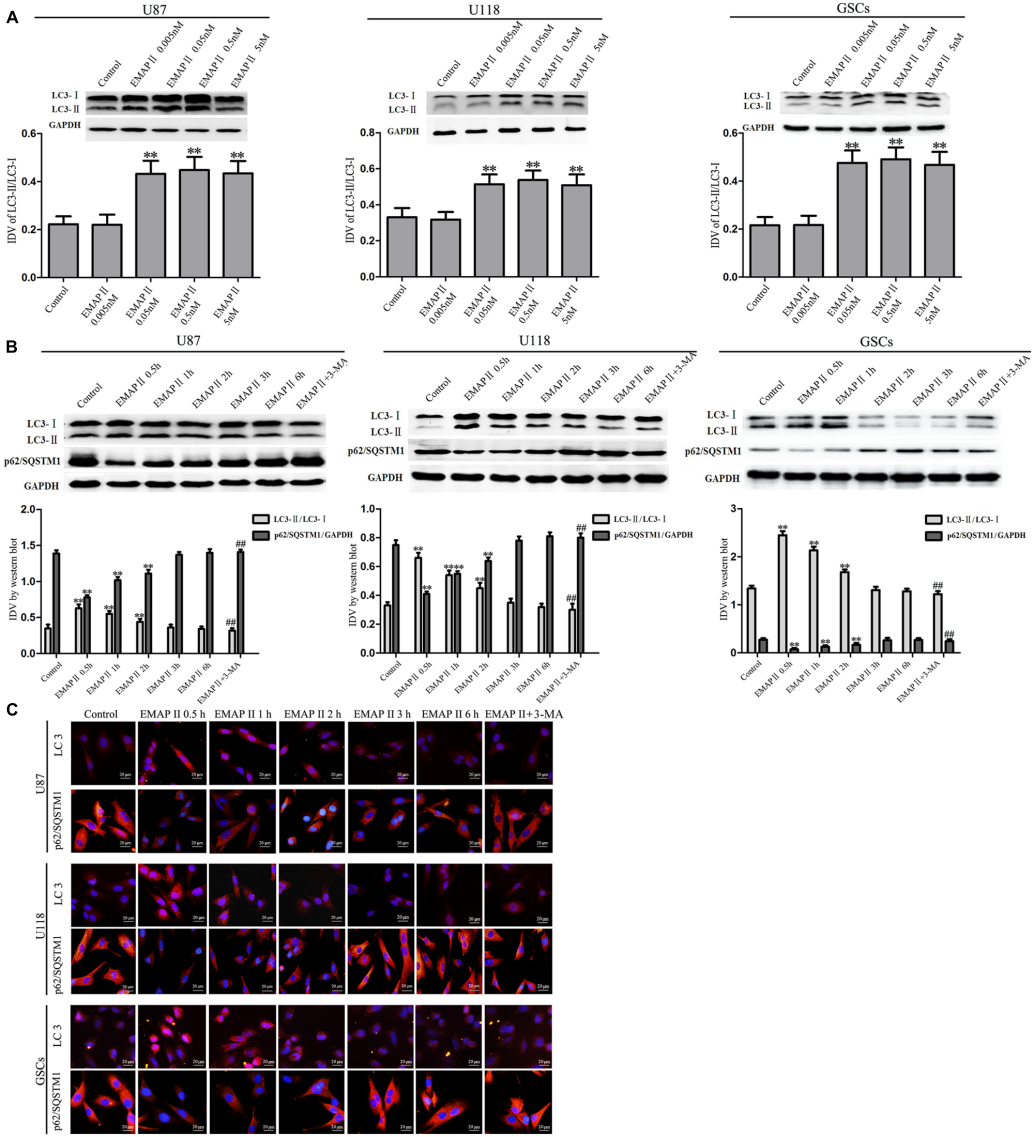
**Effects of EMAP II on the expression and distribution of LC3 and p62/SQSTM1 in human GBM cells and GSCs. (A)** Western blot analysis was performed to detect the expression levels of LC3-II/LC3-I in U87, U118, and GSCs after treatment with 0.005 nM, 0.05 nM, 0.5 nM, and 5 nM EMAP II. Relative integrated density value (IDV) of LC3-II/LC3-I are shown. **(B)** Western blot analysis was performed to detect the expression of LC3-II/LC3-I and p62/SQSTM1 in U87, U118, and GSCs after treatment with 0.05 nM EMAP II for 0.5, 1, 2, 3, and 6 h, and EMAP II pretreatment with 3-MA. IDV of LC3-II/LC3-I and p62/SQSTM1 are shown. Values represented the means ± SD (*n* = 5, each). ^∗∗^*P* < 0.01 vs. control group, ^##^*P* < 0.01 vs. EMAP II group. **(C)** The up-regulation of LC3 and down-regulation of p62/SQSTM1 in U87, U118, and GSCs were observed after treated with EMAP II alone or EMAP II plus 3-MA. Scale bar = 20 μm.

Effects of EMAP II on the expression and distribution of LC3-II and p62/SQSTM1 were detected by Western blot assay and immunofluorescence assay, respectively. As shown in **Figure 3A**, Western blot assay was used to investigate the effects of EMAP II (0.005, 0.05, 0.5, and 5 nM) on the expression of LC3-I and LC3-II in U87, U118 and GSCs at 0.5 h. Compared with the control group, LC3-II/LC3-I expression significantly increased in 0.05, 0.5, and 5 nM EMAP II groups (*P* < 0.01), whereas it did not change in 0.005 nM group. There was no significant difference among these three groups (*P* > 0.05). Further, cells were treated with EMAP II (0.05 nM) at indicated time and pretreated with 3-MA to examine the LC3 and p62/SQSTM1 expression. As shown in **Figure 3B**, LC3-II/LC3-I expression was significantly increased at 0.5, 1, and 2 h after 0.05 nM EMAP II treatment compared with the control group (*P* < 0.01). In addition, LC3-II/LC3-I expression was decreased in EMAPII+3-MA group compared with EMAP II group (*P* < 0.01), suggesting that 3-MA blocked the effect of EMAP II on the LC3-II/LC3-I expression. Meanwhile, the p62/SQSTM1 expression was significantly decreased at 0.5, 1, and 2 h after 0.05 nM EMAP II treatment (*P* < 0.01). In addition, the p62/SQSTM1 expression was increased in EMAPII+3-MA group compared with EMAP II group (*P* < 0.01), suggesting that 3-MA blocked the inhibitory effect of EMAP II on the p62/SQSTM1 expression. The immunofluorescence assay of LC3-II and p62/SQSTM1 displayed similar results as above (**Figure 3C**). These results suggested that EMAP II induced cell autophagy by regulating the expression of LC3 and p62/SQSTM1 in human GBM cells and GSCs.

### EMAP II Blocked the PI3K/Akt/mTOR Pathway in Human GBM Cells and GSCs

**FIGURE 4:**
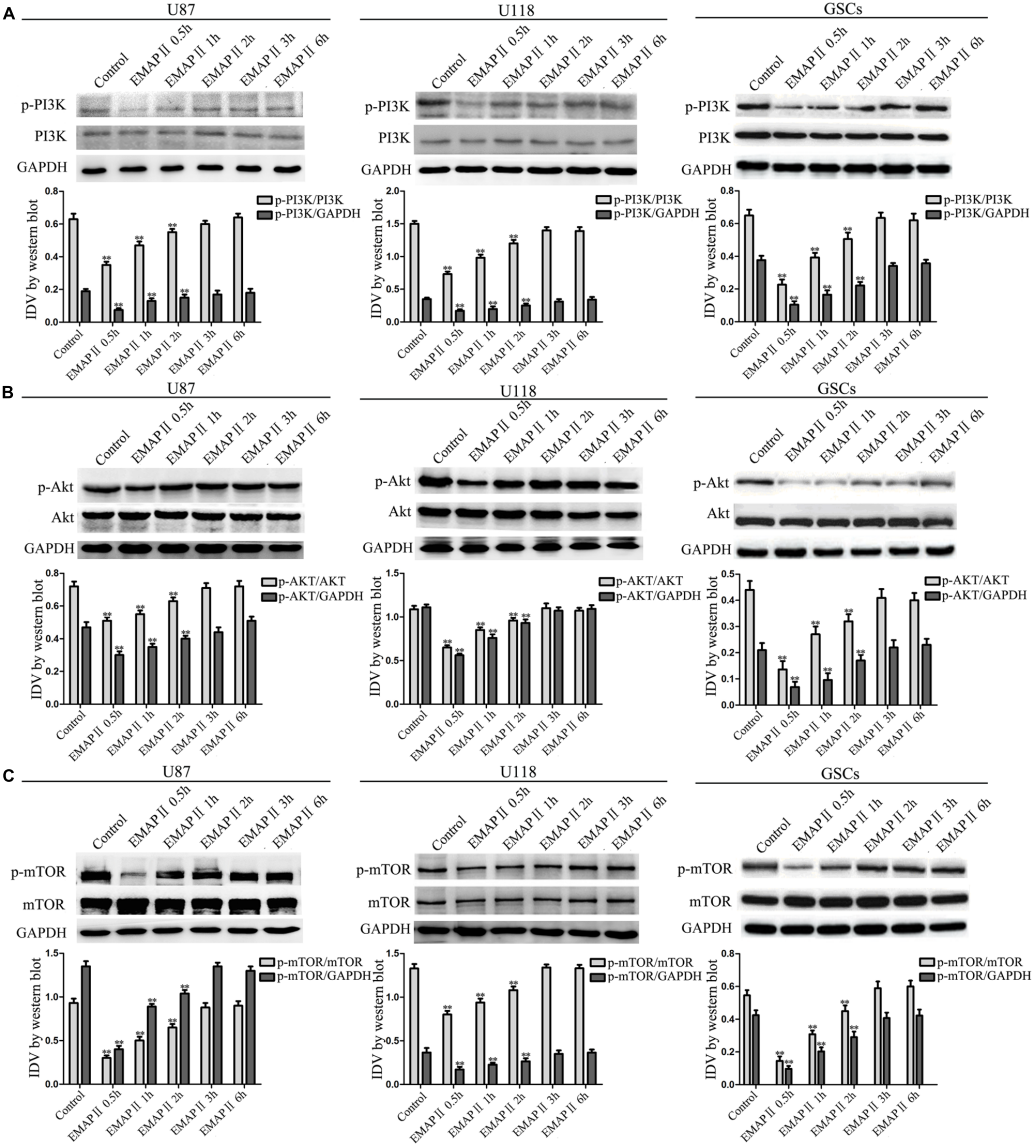
**Effect of EMAP II on the expression of mTOR, PI3K, and Akt in human GBM cells and GSCs**. Western blot analysis was performed to evaluate the expression ratios of p-PI3K/PI3K, p-PI3K/GAPDH **(A)**, p-Akt/Akt, p-Akt/GAPDH **(B)**, p-mTOR/mTOR, and p-mTOR/GAPDH **(C)** in U87, U118, and GSCs treated with EMAP II for 0.5, 1, 2, 3, and 6 h. Values represented the means ± SD (*n* = 5, each). ^∗∗^*P* < 0.01 vs. control group.

**FIGURE 5:**
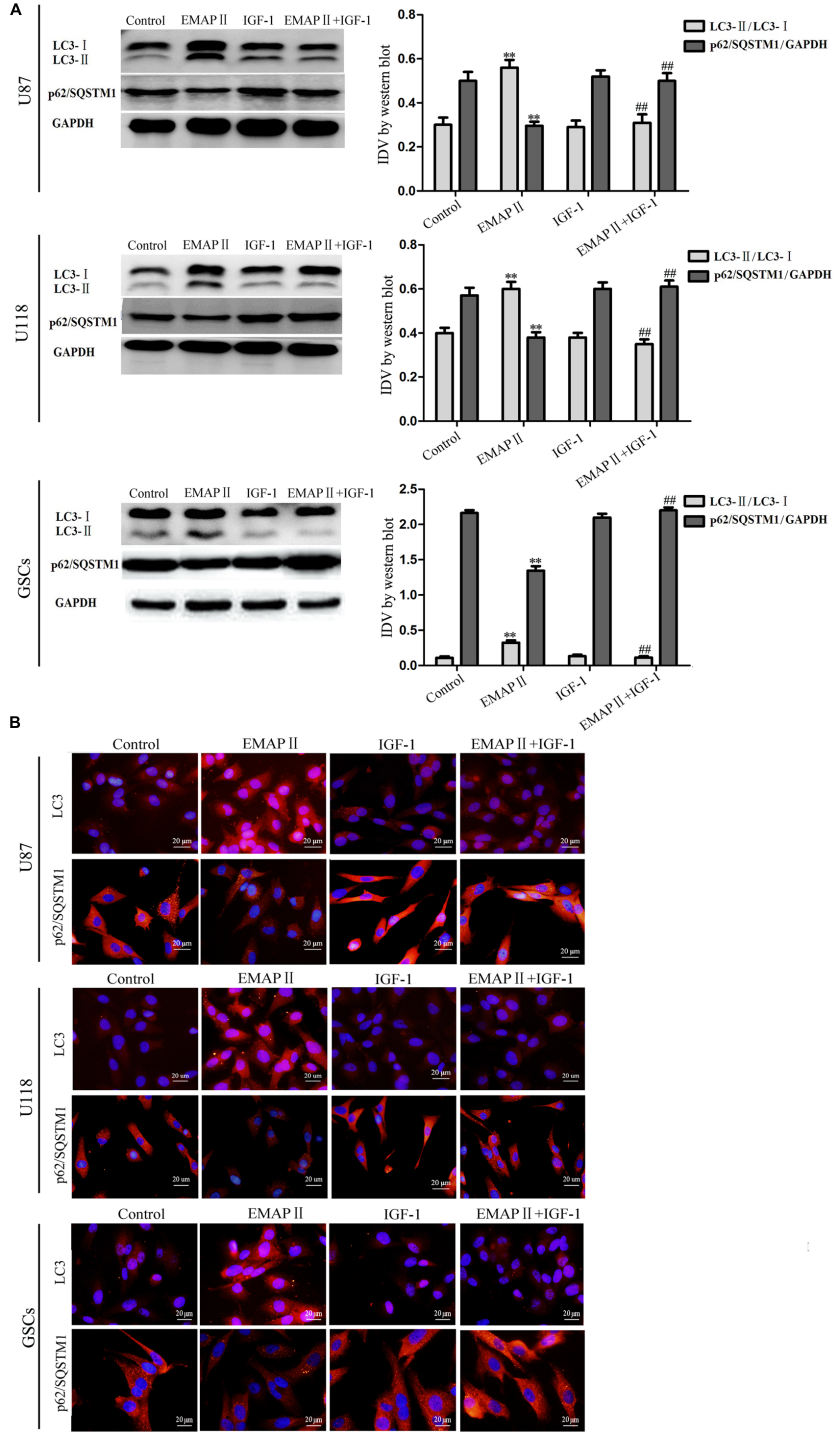
**Effects of EMAP II and IGF-1 on the expression of LC3 and p62/SQSTM1 in human GBM cells and GSCs. (A)** Western blot analysis of the expression levels of LC3-II/LC3-I and p62/SQSTM1 in U87, U118, and GSCs after treatment with EMAP II, IGF-1 and their combination. IDV of LC3-II/LC3-I and p62/SQSTM1 are shown. Values represented the means ± SD (*n* = 5, each). ^∗∗^*P* < 0.01 vs. control group, ^##^*P* < 0.01 vs. EMAP II group. **(B)** The distribution and expression of LC3 and p62/SQSTM1 in U87, U118, and GSCs. Scale bar = 20 μm.

As shown in **Figure 4A**, the expression of p-PI3K/PI3K and p-PI3K/GAPDH were decreased in U87, U118, and GSCs at 0.5, 1, and 2 h after 0.05 nM EMAP II treatment compared with the control group (*P* < 0.01), and the lowest points were at 0.5 h. The expression of p-Akt/Akt, p-Akt/GAPDH, p-mTOR/mTOR, and p-mTOR/GAPDH exhibited similar results as above (**Figures 4B,C**). As shown in **Figure 5A**, compared with the control group, cells treated with 0.05 nM EMAP II at 0.5 h significantly up-regulated the LC3-II expression and down-regulated the p62/SQSTM1 expression in U87, U118, and GSCs (*P* < 0.01). In addition, the LC3-II expression in EMAP II+IGF-1 group was decreased, whereas the p62/SQSTM1 expression in this group was increased compared with EMAP II group (*P* < 0.01). These results suggested that IGF-1 could block the effect of EMAP II on the expression of LC3-II and p62/SQSTM1. Moreover, results from immunofluorescence assay were consistent with the above results (**Figure 5B**).

### EMAP II Induced Mitophagy in Human GBM Cells and GSCs

**FIGURE 6:**
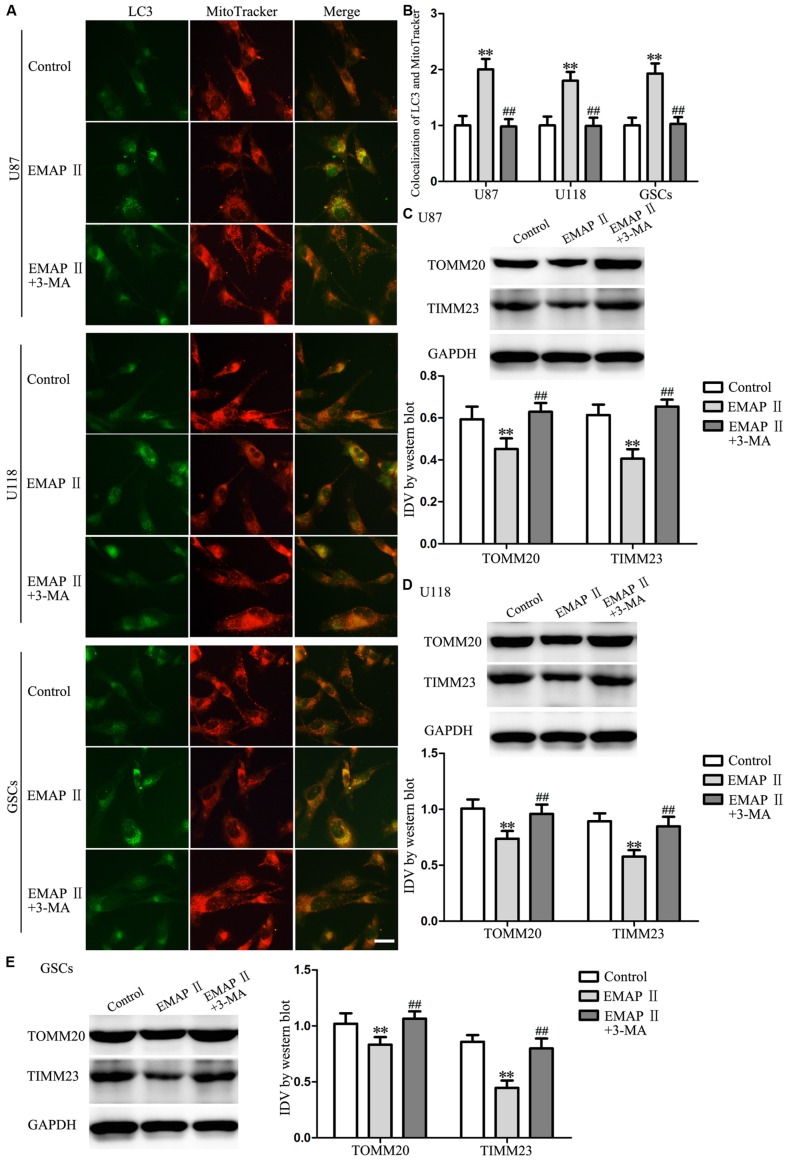
**EMAP II induced mitophagy in human GBM cells and GSCs. (A)** The colocalization of LC3 and MitoTracker in U87, U118, and GSCs after cells treated with EMAP II and the combination of EMAP II and 3-MA. **(B)** Quantitative analysis of the co-localization of LC3 with MitoTracker. Pictures are respective magnification (*n* = 4, each). Scale bar = 20 μm. Western blot analysis of the expression of TOMM20 and TIMM23 in U87 **(C)**, U118 **(D)** and GSCs **(E)** after cells treated with EMAP II and the combination of EMAP II and 3-MA. Values represented the means ± SD (*n* = 5, each). ^∗∗^*P* < 0.01 vs. control group, ^##^*P* < 0.01 vs. EMAP II group.

Due to the MMP was affected by EMAP II, therefore we detected whether mitophagy was induced by EMAP II. As shown in **Figures 6A,B**, cells were stained with MitoTracker Deep Red FM and anti-LC3 by immunofluorescence, and results showed that there was a significant overlap between LC3 and MitoTracker signals in EMAP II treated cells (*P* < 0.01), and co-treatment with 3-MA blocked this effect (*P* < 0.01). In addition, the outer and inner mitochondrial membrane proteins (TOMM20 and TIMM23) was reduced by EMAP II, and co-treatment with 3-MA rescued the inhibitory effect induced by EMAP II (**Figures 6C–E**).

### EMAP-II Induced the Activation of Endoplasmic Reticulum (ER) Stress in Human GBM Cells and GSCs

**FIGURE 7:**
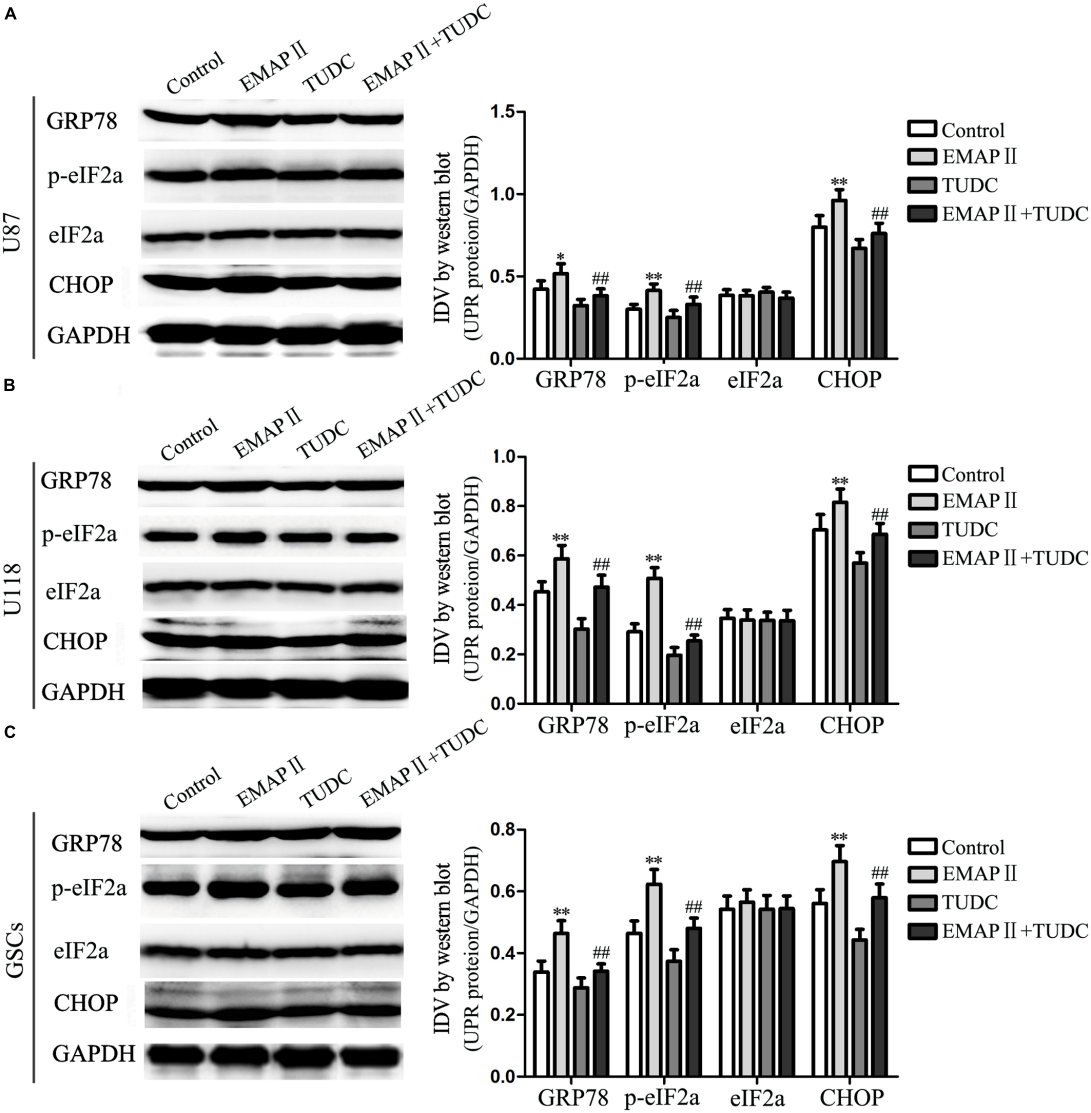
**Effects of EMAP II and tauroursodeoxycholate (TUDC) on the expression of UPR-related proteins in human GBM cells and GSCs**. Western blot analysis of the expression of GRP78, p-eIF2α, eIF2α, and CHOP in U87 **(A)**, U118 **(B),** and GSCs **(C)** after treatment with EMAP II, TUDC and their combination. Values represented the means ± SD (*n* = 5, each). ^∗^*P* < 0.05 and ^∗∗^*P* < 0.01 vs. control group, ^##^*P* < 0.01 vs. EMAP II group.

Several studies reported that autophagy could be induced by unfolded protein response (UPR) which is the major endoplasmic reticulum (ER) stress pathway (Tsai and Weissman, 2010). To examine whether ER stress was induced by the EMAP-II treatment, levels of the UPR-related proteins GRP78, p-eIF2α, eIF2α, and CHOP were measured after cells treated with 0.05 nM EMAP II for 0.5 h, (TUDC, an ER stress inhibitor) or their combination. As shown in **Figure 7A**, the expression of GRP78, p-eIF2α, and CHOP were increased in EMAP II group compared with the control group in U87 cells (*P* < 0.01). In addition, the expression of GRP78, p-eIF2α, and CHOP were decreased in the combination of EMAP II with TUDC group compared with the EMAP II group (*P* < 0.01). The similar results were also observed in U118 cells and GSCs (**Figures 7B,C**). These results suggested that human GBM cells and GSCs exposed to EMAP-II experienced ER stress.

### The Combination of EMAP II with Rapamycin Inhibited Cell Proliferation, Migration, and Invasion of Human GBM Cells and GSCs

**FIGURE 8:**
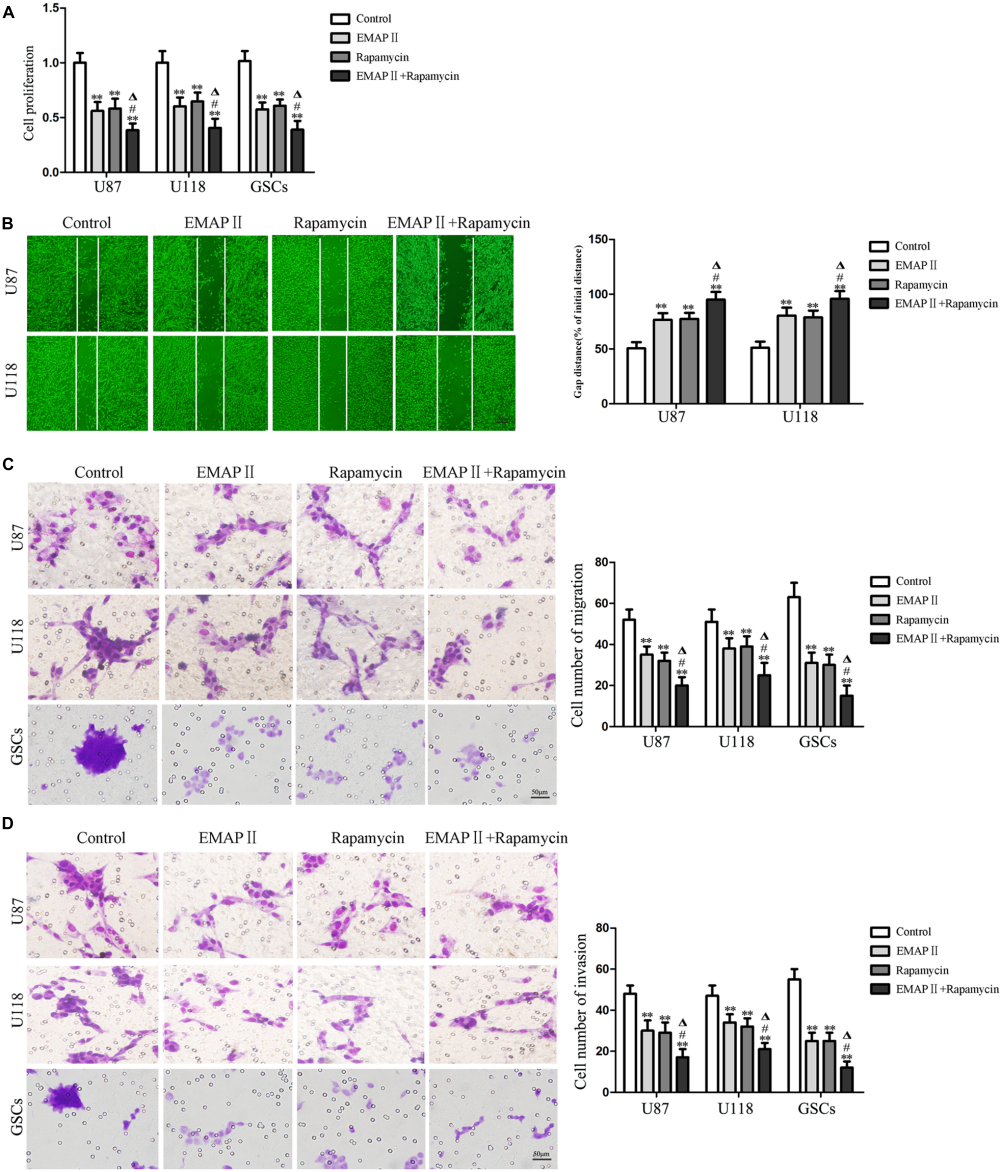
**Effects of EMAP II and rapamycin on the cell proliferation, migration, and invasion of human GBM cells and GSCs. (A)** Cell proliferation of U87, U118, and GSCs was assessed by CCK8 assay. **(B)** The migration of U87 and U118 cells was measured by wound healing assay. **(C)** Cell migration of U87, U118, and GSCs was measured by transwell assay. **(D)** Cell invasion of U87, U118, and GSCs was measured by transwell assay. Values represented the means ± SD (*n* = 5, each). ^∗∗^*P* < 0.01 vs. control group; ^#^*P* < 0.05 vs. EMAP II group; ^Δ^*P* < 0.05 vs. rapamycin group.

The abilities of cell proliferation, migration and invasion were investigated to further evaluate the effects of the combination of EMAP II with rapamycin on the biological behaviors of human GBM cells and GSCs. As shown in **Figure 8A**, compared with the control group, the cell proliferation of U87, U118, and GSCs were inhibited after EMAP II or rapamycin treatment (*P* < 0.01). The cell proliferation of U87, U118, and GSCs was more remarkably inhibited by the combination of EMAP II with rapamycin compared with the use of either agent alone (*P* < 0.05). As shown in **Figure 8B**, scratch wound healing assay showed that the migration of U87 and U118 cells in EMAP II or rapamycin groups were inhibited compared with that in the control group (*P* < 0.01). In addition, EMAP II in combination with rapamycin displayed even greater inhibitory effect on the migration of U87 and U118 cells than either EMAP II or rapamycin alone (*P* < 0.05). Subsequently, we employed the transwell migration assay to further define the migration of U87, U118 and GSCs, and the results were similar as above (**Figure 8C**). As shown in **Figure 8D**, the invasion of U87, U118, and GSCs in EMAP II or rapamycin groups were inhibited compared with that in the control group (*P* < 0.01). EMAP II in combination with rapamycin displayed even greater inhibitory effect on the invasion of U87, U118, and GSCs than either EMAP II or rapamycin alone (*P* < 0.05). The above results suggested that the combination of EMAP II with rapamycin inhibited the malignant biological behaviors of human GBM cells and GSCs.

### The Combination of EMAP II with Rapamycin Produced the Smallest Tumors *In Vivo*

**FIGURE 9:**
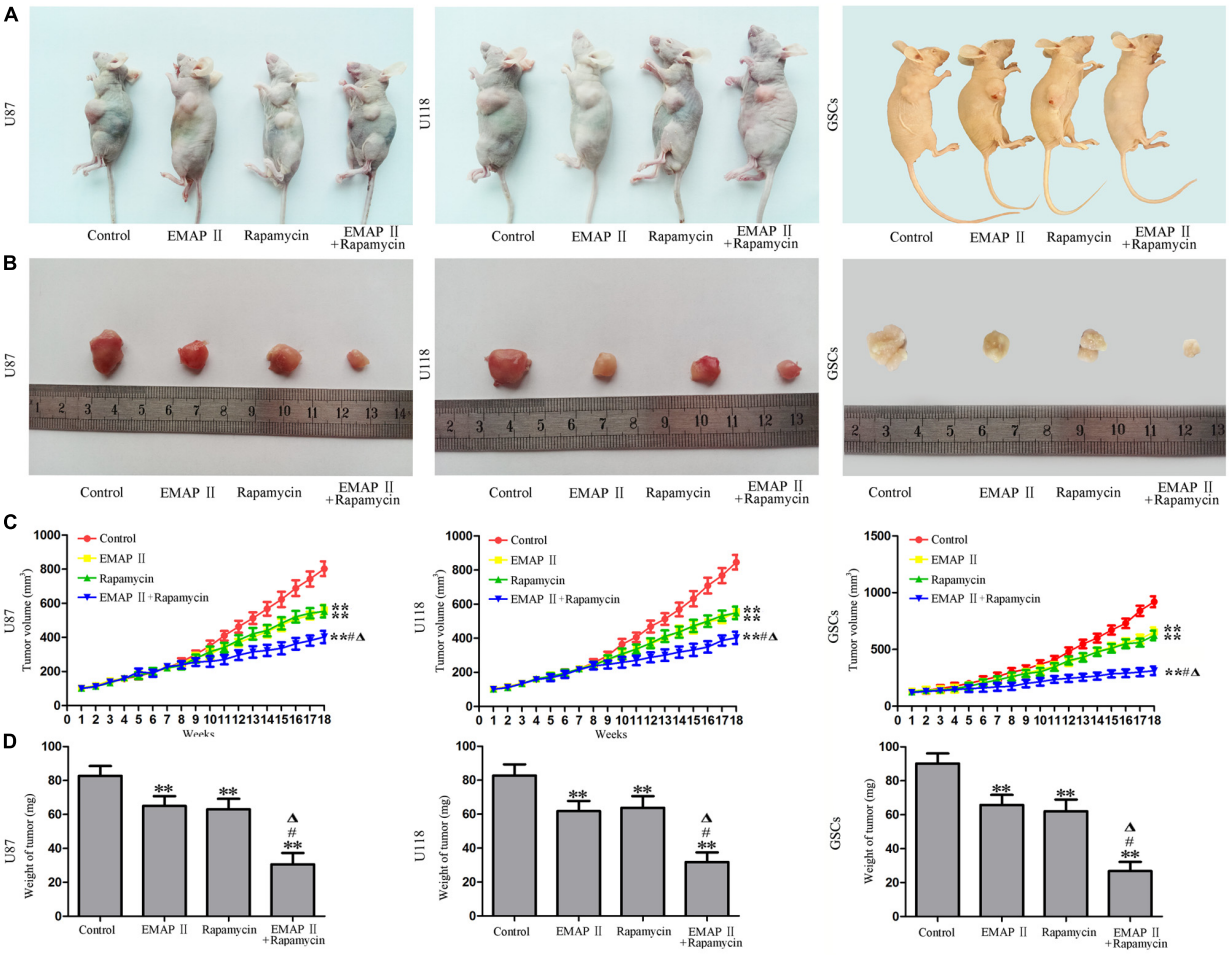
**The *in vivo* study of EMAP II and rapamycin**. Representative images of mice **(A)** and tumors **(B)** obtained from the xenografted mice with or without treatment for 18 weeks. **(C)** Tumor growth curve in nude mice. **(D)** Tumor weight in nude mice. ^∗∗^*P* < 0.01 vs. control group, ^#^*P* < 0.05 vs. EMAP-II group, ^Δ^*P* < 0.05 vs. rapamycin group.

The growth-inhibitory effects of EMAP-II and rapamycin were further tested in xenografted mice. Representative images of mice and tumors were shown in **Figures 9A,B**. Tumor growth was significantly suppressed in nude mice treated with EMAP-II, rapamycin or the combination of EMAP II with rapamycin compared with that in the control group (*P* < 0.01). Combined treatment of EMAP II with rapamycin produced further inhibition on tumor growth than EMAP-II or rapamycin group (*P* < 0.05) (**Figures 9C,D**). These results showed that nude mice carrying human GBM cells and GSCs which were treated with the combination of EMAP II with rapamycin produced the smallest tumors.

## Discussion

In the present study, we found that low-dose EMAP II inhibited the cell viability and MMP in human GBM cells and GSCs by inducing autophagy, accompanied by the up-regulation of LC3-II and the down-regulation of p62/SQSTM1. Moreover, we provided the evidence that cells treated with EMAP-II inhibited the PI3K/Akt/mTOR signal pathway, and experienced mitophagy and ER stress. Finally, the combination of EMAP II and rapamycin showed the inhibitory effect on the cell proliferation, migration, and invasion of GBM cells and GSCs *in vitro* and *in vivo*.

Endothelial-monocyte activating polypeptide II has been recognized as an angiostatic mediator that suppresses neovascularization, induced endothelial cell apoptosis, inhibited tumor vessel formation, and suppressed primary and metastatic tumor growth (Schwarz et al., 1999; Berger et al., 2000). In addition, EMAP II was found to reduce the proliferation of tumor cells within the neoplastic tissue (Schwarz and Schwarz, 2004). Our results showed that low-dose EMAP II significantly inhibited the cell viability of human GBM cells and GSCs at 0.5 h. To further analyze the possible mechanisms, cells were pretreated with autophagy inhibitor 3-MA or broad-spectrum caspase inhibitor Z-VAD. Our results showed that only 3-MA blocked the inhibitory effect of EMAP II on the cell viability. It has been reported that EMAP II inhibited the growth of endothelial cells and pancreatic cancer cells, and the inhibitory effect was associated with the inhibition of tumor vascular endothelium growth (Schwarz and Schwarz, 2004). However, the underlying mechanism in regarding to this inhibition may involve many aspects, such as EMAP II inhibited the growth of pancreatic cancer through inhibiting fibronectin-dependent proliferation (Schwarz et al., 2010a,b) or inhibited tumor angiogenesis by inducing endothelial cell apoptosis (Berger et al., 2000). However, the dose of EMAP II described in our present study has not been reported to induce apoptosis in GBM cells and GSCs. Mitochondrial functional status is closely related to cancerogenesis, and MMP is one of the important parameters of mitochondrial functional status. Our results showed that EMAP II decreased the MMP and formed a trough at 0.5 h. Meanwhile, EMAP II-induced MMP inhibition was blocked by 3-MA, indicating that EMAP II inhibited cell viability and impaired the mitochondrial function might be associated with autophagy. Additionally, we observed the existence of autophgosomes and the increased punctate distribution of LC3 under the treatment of 0.05 nM EMAP II for 0.5 h, and 3-MA blocked this effect. Thus, our results demonstrated that the cytotoxicity of EMAP II in human GBM cells and GSCs was induced by autophagy. Moreover, mitochondria have been described as a source of autophagosome biogenesis, and plays a key role in the cross-talk between autophagy and apoptosis regulation (Hailey et al., 2010; Strappazzon et al., 2011). Mitophagy is a process through which dysfunctional mitochondria are selectively removed by autophagy (Kim et al., 2007). Our results showed that EMAP II induced mitophagy in human GBM cells and GSCs, suggesting that the mitophagy might be involved in the cytotoxicity induced by EMAP II.

LC3, an autophagy marker, exists in cells in two forms: LC3-I and LC3-II. As a marker of autophgosomes, the expression level of LC3-II reflects the number of autophgosomes to a certain extent (Levine and Yuan, 2005). Our study revealed that EMAP II significantly increased LC3-II expression at the concentrations of 0.05, 0.5, and 5 nM. The expression of LC3-II was increased to peak level at 0.5 h under the treatment of 0.05 nM EMAP II with the punctuate of LC3 aggregated in GBM cells and GSCs. P62/SQSTM1 is a multifunctional ubiquitinated protein coupled to LC3, which is involved in the formation of autophgosomes as a regulatory factor and is degraded in the middle or late phase of autophagy. Therefore, the total expression level of intracellular p62/SQSTM1 was negatively correlated with autophagic activity (Bjorkoy et al., 2005). Consistent with the above study, the effect of EMAP II on the p62/SQSTM1 expression was opposite to LC3-II. P62/SQSTM1 plays important roles in the regulation of intracellular autophagy, proteasome and NF-κB signaling pathway. It has been reported that the repression of autophagy led to over-expression of p62/SQSTM1, which promoted tumor cell proliferation by various mechanisms such as regulating NF-κB signaling pathway, the accumulation of oxygen radicals, DNA damage and other processes (Kongara and Karantza, 2012; Moscat and Diaz-Meco, 2012).

PI3K/Akt/mTOR signal pathway is mainly involved in regulating cell growth, apoptosis and other important cellular signal transduction (Cherla et al., 2009; Zhang et al., 2009). Furthermore, PI3K/Akt/mTOR is a critical regulatory pathway in autophagy response (Takeuchi et al., 2005; Guertin and Sabatini, 2007). Recent study revealed that PI3K/Akt/mTOR signal pathway would be commonly activated in human glioma cells (Qiao et al., 2013; Shin et al., 2013; Xu et al., 2013). To further investigate the possible mechanism involved in this process, we studied the effect of EMAP II on P13K, Akt, and mTOR signal molecules. Our study found that the PI3K/Akt/mTOR signal pathway was inhibited at 0.5, 1, and 2 h after 0.05 nM EMAP II treatment, and the PI3K/Akt agonist IGF-1 significantly blocked the effect of EMAP II on the expression of LC3-II and p62/SQSTM1, suggesting the EMAP-II-induced autophagy is dependent of PI3K/Akt/mTOR signal pathway. The effect of some anti-neoplastic drugs are also associated with the regulation of PI3K/Akt/mTOR signal pathway. Chlorpromazine was reported to induce autophagic cell death in U87 cells through inhibiting Akt/mTOR (Shin et al., 2013). The inhibition of mTOR cascade reaction pathway was also involved in the synergistic anti-glioma effect of niclosamide in combination with emozolomide (Takeuchi et al., 2005).

The endoplasmic reticulum (ER) is responsible for protein folding, lipid and sterol biosynthesis, and intracellular calcium storage (Schroder, 2008). Accumulation of unfolded proteins in the ER, nutrient deprivation, calcium imbalance, oxidative stress, metabolic alterations, and environmental acidity activate UPR signaling to enable adaptation to perturbations and to relieve ER stress by activating three ER-resident transmembrane transducers (Kim et al., 2008; Cao and Kaufman, 2012). Autophagy could supplement cellular metabolism by digesting damaged organelles or misfolded proteins. In addition, recent studies have confirmed that ER stress is a potent inducer of autophagy (Cook et al., 2014; Shen et al., 2014), thus the levels of the UPR-related proteins GRP78, p-eIF2α, eIF2α, and CHOP were measured. Our results showed that the expression of GRP78, p-eIF2α, and CHOP were increased in EMAP II group, TUDC attenuated the expression of these induced by EMAP II, suggesting that cells exposed to EMAP-II experienced ER stress. In addition, we have demonstrated that EMAP II induced cell autophagy in human GBM cells and GSCs, suggesting that autophagy induced by EMAP-II might be related with ER stress.

Rapamycin, an mTOR inhibitor, is widely used in the experimental and clinical researches for anti-neoplastic drugs (Nakamura et al., 2014; Wang et al., 2014). The mTOR inhibitors have been used in the clinical trial for several tumors, among them are rapamycin analogs temsirolimus and everolimus which have been used to treat some tumors (Leung et al., 2011; Fasolo and Sessa, 2012; Voss et al., 2014). In the study of glioma treatment, everolimus led to cell cycle arrest and increased radiosensitivity in malignant glioma cells by activating autophagy response (Scaringi et al., 2013). Therefore, we further evaluated the effects of EMAP II alone or in combination with rapamycin on the biological behaviors of human GBM cells and GSCs. Our results showed that both EMAP II and rapamycin significantly inhibited the cell proliferation, migration, and invasion of human GBM cells and GSCs. The combination of these two agents displayed more inhibitory effect on the malignant behavior of these cells. The *in vivo* studies also showed that the combination of EMAP II with rapamycin produced the smallest tumors. In line with our study, previous study have shown that rapamycin could inhibit the proliferation of glioma cells by inducing autophagy (Bray et al., 2012), which contributed to the anti-glioma effect of EMAP II combined with rapamycin.

As mentioned above, our study illustrated that the cytotoxicity of low-dose EMAP II in human GBM cells and GSCs was induced by autophagy, accompanied by the inhibition of PI3K/Akt/mTOR signal pathway, mitophagy and ER stress. Furthermore, EMAP II combined with rapamycin significantly inhibited cell proliferation, migration and invasion of human GBM cells and GSCs. Thus, the combined therapy of EMAP II with agents that can inhibit P13K/Akt/mTOR signal pathway may be a new novel therapeutic strategy for the treatment of human glioblastoma.

## Author Contributions

Conceived and designed the experiments: YX, YL, and LL. Performed the experiments: JM, FM, SL, and LL. Analyzed the data: LZ, YH, ZL, and YY. Contributed reagents/materials/analysis tools: ZX, HT, and YL. Wrote the manuscript: JM, FM, SL, and YX.

## Conflict of Interest Statement

The authors declare that the research was conducted in the absence of any commercial or financial relationships that could be construed as a potential conflict of interest.

## References

[B1] Al-Ejeh F. Kumar R. Wiegmans A. Lakhani S. R. Brown M. P. Khanna K. K. (2010). Harnessing the complexity of DNA-damage response pathways to improve cancer treatment outcomes. *Oncogene* 29 6085–6098. 10.1038/onc.2010.407 20818418

[B2] Awasthi N. Schwarz M. A. Schwarz R. E. (2010). Combination effects of bortezomib with gemcitabine and EMAP II in experimental pancreatic cancer. *Cancer Biol. Ther.* 10 99–107. 10.4161/cbt.10.1.12169 20495354

[B3] Bao S. Wu Q. McLendon R. E. Hao Y. Shi Q. Hjelmeland A. B. (2006). Glioma stem cells promote radioresistance by preferential activation of the DNA damage response. *Nature* 444 756–760. 10.1038/nature05236 17051156

[B4] Berger A. C. Alexander H. R. Tang G. Wu P. S. Hewitt S. M. Turner E. (2000). Endothelial monocyte activating polypeptide II induces endothelial cell apoptosis and may inhibit tumor angiogenesis. *Microvasc. Res.* 60 70–80. 10.1006/mvre.2000.2249 10873516

[B5] Bjorkoy G. Lamark T. Brech A. Outzen H. Perander M. Overvatn A. (2005). p62/SQSTM1 forms protein aggregates degraded by autophagy and has a protective effect on huntingtin-induced cell death. *J. Cell Biol.* 171 603–614. 10.1083/jcb.200507002 16286508 PMC2171557

[B6] Bray K. Mathew R. Lau A. Kamphorst J. J. Fan J. Chen J. (2012). Autophagy suppresses RIP kinase-dependent necrosis enabling survival to mTOR inhibition. *PLoS ONE* 7:e41831 10.1371/journal.pone.0041831 PMC3406086 22848625

[B7] Cao S. S. Kaufman R. J. (2012). Unfolded protein response. *Curr. Biol.* 22 R622–R626. 10.1016/j.cub.2012.07.004 22917505

[B8] Chen N. Karantza-Wadsworth V. (2009). Role and regulation of autophagy in cancer. *Biochim. Biophys. Acta* 1793 1516–1523. 10.1016/j.bbamcr.2008.12.013 19167434 PMC3155287

[B9] Cherla R. P. Lee S. Y. Mulder R. A. Lee M. S. Tesh V. L. (2009). Shiga toxin 1-induced proinflammatory cytokine production is regulated by the phosphatidylinositol 3-kinase/Akt/mammalian target of rapamycin signaling pathway. *Infect. Immun.* 77 3919–3931. 10.1128/IAI.00738-739. 19596774 PMC2737994

[B10] Cook K. L. Clarke P. A. Parmar J. Hu R. Schwartz-Roberts J. L. Abu-Asab M. (2014). Knockdown of estrogen receptor-alpha induces autophagy and inhibits antiestrogen-mediated unfolded protein response activation, promoting ROS-induced breast cancer cell death. *FASEB J.* 28 3891–3905. 10.1096/fj.13-247353 24858277 PMC4139896

[B11] Faisal W. Symonds P. Panjwani S. Heng Y. Murray J. C. (2007). Cell-surface associated p43/endothelial-monocyte-activating-polypeptide-II in hepatocellular carcinoma cells induces apoptosis in T-lymphocytes. *Asian J. Surg.* 30 13–22. 10.1016/S1015-9584(09)60122-60126 17337366

[B12] Fasolo A. Sessa C. (2012). Targeting mTOR pathways in human malignancies. *Curr. Pharm. Des.* 18 2766–2777. 10.2174/138161212800626210 22475451

[B13] Fu J. Shao C. J. Chen F. R. Ng H. K. Chen Z. P. (2010). Autophagy induced by valproic acid is associated with oxidative stress in glioma cell lines. *Neuro Oncol.* 12 328–340. 10.1093/neuonc/nop005 20308311 PMC2940599

[B14] Guertin D. A. Sabatini D. M. (2005). An expanding role for mTOR in cancer. *Trends Mol. Med.* 11 353–361. 10.1016/j.molmed.2005.06.007 16002336

[B15] Guertin D. A. Sabatini D. M. (2007). Defining the role of mTOR in cancer. *Cancer Cell* 12 9–22. 10.1016/j.ccr.2007.05.008 17613433

[B16] Hadjipanayis C. G. Van Meir E. G. (2009). Brain cancer propagating cells: biology, genetics and targeted therapies. *Trends Mol. Med.* 15 519–530. 10.1016/j.molmed.2009.09.003 19889578 PMC2782740

[B17] Hailey D. W. Rambold A. S. Satpute-Krishnan P. Mitra K. Sougrat R. Kim P. K. (2010). Mitochondria supply membranes for autophagosome biogenesis during starvation. *Cell* 141 656–667. 10.1016/j.cell.2010.04.009 20478256 PMC3059894

[B18] Kanzawa T. Germano I. M. Komata T. Ito H. Kondo Y. Kondo S. (2004). Role of autophagy in temozolomide-induced cytotoxicity for malignant glioma cells. *Cell Death Differ.* 11 448–457. 10.1038/sj.cdd.4401359 14713959

[B19] Kao J. Houck K. Fan Y. Haehnel I. Libutti S. K. Kayton M. L. (1994). Characterization of a novel tumor-derived cytokine. Endothelial-monocyte activating polypeptide II. *J. Biol. Chem.* 269 25106–25119. 7929199

[B20] Kim I. Rodriguez-Enriquez S. Lemasters J. J. (2007). Selective degradation of mitochondria by mitophagy. *Arch. Biochem. Biophys.* 462 245–253. 10.1016/j.abb.2007.03.034 17475204 PMC2756107

[B21] Kim I. Xu W. Reed J. C. (2008). Cell death and endoplasmic reticulum stress: disease relevance and therapeutic opportunities. *Nat. Rev. Drug Discov.* 7 1013–1030. 10.1038/nrd2755 19043451

[B22] Kongara S. Karantza V. (2012). The interplay between autophagy and ROS in tumorigenesis. *Front. Oncol.* 2:171 10.3389/fonc.2012.00171 PMC3502876 23181220

[B23] Leung E. Kim J. E. Rewcastle G. W. Finlay G. J. Baguley B. C. (2011). Comparison of the effects of the PI3K/mTOR inhibitors NVP-BEZ235 and GSK2126458 on tamoxifen-resistant breast cancer cells. *Cancer Biol. Ther.* 11 938–946. 10.4161/cbt.11.11.15527 21464613 PMC3127046

[B24] Levine B. Yuan J. (2005). Autophagy in cell death: an innocent convict? *J. Clin. Invest.* 115 2679–2688. 10.1172/JCI26390 16200202 PMC1236698

[B25] Li Z. Liu X. B. Liu Y. H. Xue Y. X. Wang P. Liu L. B. (2015). Functions for the cAMP/Epac/Rap1 signaling pathway in low-dose endothelial monocyte-activating polypeptide-II-induced opening of blood-tumor barrier. *J. Mol. Neurosci.* 57 1–10. 10.1007/s12031-015-0594-596 26044663

[B26] Li Z. Liu Y. H. Xue Y. X. Liu L. B. Wang P. (2012). Signal mechanisms underlying low-dose endothelial monocyte-activating polypeptide-II-induced opening of the blood-tumor barrier. *J. Mol. Neurosci.* 48 291–301. 10.1007/s12031-012-9776-9777 22531886

[B27] Minniti G. De Sanctis V. Muni R. Filippone F. Bozzao A. Valeriani M. (2008). Radiotherapy plus concomitant and adjuvant temozolomide for glioblastoma in elderly patients. *J. Neurooncol.* 88 97–103. 10.1007/s11060-008-9538-0 18250965

[B28] Moscat J. Diaz-Meco M. T. (2012). p62: a versatile multitasker takes on cancer. *Trends Biochem. Sci.* 37 230–236. 10.1016/j.tibs.2012.02.008 22424619 PMC3531712

[B29] Murray J. C. Heng Y. M. Symonds P. Rice K. Ward W. Huggins M. (2004a). Endothelial monocyte-activating polypeptide-II (EMAP-II): a novel inducer of lymphocyte apoptosis. *J. Leukoc. Biol.* 75 772–776. 10.1189/jlb.1003487 14982944

[B30] Murray J. C. Symonds P. Ward W. Huggins M. Tiga A. Rice K. (2004b). Colorectal cancer cells induce lymphocyte apoptosis by an endothelial monocyte-activating polypeptide-II-dependent mechanism. *J. Immunol.* 172 274–281. 10.4049/jimmunol.172.1.274 14688335

[B31] Nabissi M. Morelli M. B. Amantini C. Liberati S. Santoni M. Ricci-Vitiani L. (2015). Cannabidiol stimulates Aml-1a-dependent glial differentiation and inhibits glioma stem-like cells proliferation by inducing autophagy in a TRPV2-dependent manner. *Int. J. Cancer* 137 1855–1869. 10.1002/ijc.29573 25903924

[B32] Nakamura O. Hitora T. Yamagami Y. Mori M. Nishimura H. Horie R. (2014). The combination of rapamycin and MAPK inhibitors enhances the growth inhibitory effect on Nara-H cells. *Int. J. Mol. Med.* 33 1491–1497. 10.3892/ijmm.2014.1715 24676456 PMC4055350

[B33] Omuro A. DeAngelis L. M. (2013). Glioblastoma and other malignant gliomas: a clinical review. *JAMA* 310 1842–1850. 10.1001/jama.2013.280319 24193082

[B34] Piccirillo S. G. Binda E. Fiocco R. Vescovi A. L. Shah K. (2009). Brain cancer stem cells. *J. Mol. Med. (Berl.)* 87 1087–1095. 10.1007/s00109-009-0535-533 19784875

[B35] Qiao D. Meyer K. Friedl A. (2013). Glypican 1 stimulates s phase entry and DNA replication in human glioma cells and normal astrocytes. *Mol. Cell. Biol.* 33 4408–4421. 10.1128/MCB.00238-213 24019070 PMC3838173

[B36] Scaringi C. Enrici R. M. Minniti G. (2013). Combining molecular targeted agents with radiation therapy for malignant gliomas. *Onco Targets Ther.* 6 1079–1095. 10.2147/OTT.S48224 23966794 PMC3745290

[B37] Schroder M. (2008). Endoplasmic reticulum stress responses. *Cell Mol. Life. Sci.* 65 862–894. 10.1007/s00018-007-7383-5 18038217 PMC11131897

[B38] Schwarz M. Lee M. Zhang F. Zhao J. Jin Y. Smith S. (1999). EMAP II: a modulator of neovascularization in the developing lung. *Am. J. Physiol.* 276 L365–L375. 9950900 10.1152/ajplung.1999.276.2.L365

[B39] Schwarz R. E. Awasthi N. Konduri S. Cafasso D. Schwarz M. A. (2010a). EMAP II-based antiangiogenic-antiendothelial in vivo combination therapy of pancreatic cancer. *Ann. Surg. Oncol.* 17 1442–1452. 10.1245/s10434-009-0879-875 20041350

[B40] Schwarz R. E. Awasthi N. Konduri S. Caldwell L. Cafasso D. Schwarz M. A. (2010b). Antitumor effects of EMAP II against pancreatic cancer through inhibition of fibronectin-dependent proliferation. *Cancer Biol. Ther.* 9 632–639. 10.4161/cbt.9.8.11265 20212356

[B41] Schwarz R. E. Schwarz M. A. (2004). In vivo therapy of local tumor progression by targeting vascular endothelium with EMAP-II. *J. Surg. Res.* 120 64–72. 10.1016/j.jss.2003.10.005 15172191

[B42] Shen S. Zhang Y. Wang Z. Liu R. Gong X. (2014). Bufalin induces the interplay between apoptosis and autophagy in glioma cells through endoplasmic reticulum stress. *Int. J. Biol. Sci.* 10 212–224. 10.7150/ijbs.8056 24550689 PMC3927133

[B43] Shin S. Y. Lee K. S. Choi Y. K. Lim H. J. Lee H. G. Lim Y. (2013). The antipsychotic agent chlorpromazine induces autophagic cell death by inhibiting the Akt/mTOR pathway in human U-87MG glioma cells. *Carcinogenesis* 34 2080–2089. 10.1093/carcin/bgt169 23689352

[B44] Singh S. K. Clarke I. D. Terasaki M. Bonn V. E. Hawkins C. Squire J. (2003). Identification of a cancer stem cell in human brain tumors. *Cancer Res.* 63 5821–5828. 14522905

[B45] Singh S. K. Hawkins C. Clarke I. D. Squire J. A. Bayani J. Hide T. (2004). Identification of human brain tumour initiating cells. *Nature* 432 396–401. 10.1038/nature03128 15549107

[B46] Stathaki M. Armakolas A. Dimakakos A. Kaklamanis L. Vlachos I. Konstantoulakis M. M. (2014). Kisspeptin effect on endothelial monocyte activating polypeptide II (EMAP-II)-associated lymphocyte cell death and metastases in colorectal cancer patients. *Mol. Med.* 20 80–92. 10.2119/molmed.2013.00151 24395571 PMC3960398

[B47] Strappazzon F. Vietri-Rudan M. Campello S. Nazio F. Florenzano F. Fimia G. M. (2011). Mitochondrial BCL-2 inhibits AMBRA1-induced autophagy. *EMBO J.* 30 1195–1208. 10.1038/emboj.2011.49 21358617 PMC3094111

[B48] Takeuchi H. Kondo Y. Fujiwara K. Kanzawa T. Aoki H. Mills G. B. (2005). Synergistic augmentation of rapamycin-induced autophagy in malignant glioma cells by phosphatidylinositol 3-kinase/protein kinase *B inhibitors*. *Cancer Res.* 65 3336–3346. 10.1158/0008-5472.CAN-04-3640 15833867

[B49] Tanaka S. Louis D. N. Curry W. T. Batchelor T. T. Dietrich J. (2013). Diagnostic and therapeutic avenues for glioblastoma: no longer a dead end? *Nat. Rev. Clin. Oncol.* 10 14–26. 10.1038/nrclinonc.2012.204 23183634

[B50] Tsai Y. C. Weissman A. M. (2010). The unfolded protein response, degradation from endoplasmic reticulum and cancer. *Genes Cancer* 1 764–778. 10.1177/1947601910383011 21331300 PMC3039444

[B51] Visvader J. E. Lindeman G. J. (2008). Cancer stem cells in solid tumours: accumulating evidence and unresolved questions. *Nat. Rev. Cancer* 8 755–768. 10.1038/nrc2499 18784658

[B52] Voss M. H. Bastos D. A. Karlo C. A. Ajeti A. Hakimi A. A. Feldman D. R. (2014). Treatment outcome with mTOR inhibitors for metastatic renal cell carcinoma with nonclear and sarcomatoid histologies. *Ann. Oncol.* 25 663–668. 10.1093/annonc/mdt578 24458473 PMC4229900

[B53] Wang Z. Xu F. Yuan N. Niu Y. Lin W. Cao Y. (2014). Rapamycin inhibits pre-B acute lymphoblastic leukemia cells by downregulating DNA and RNA polymerases. *Leuk Res.* 38 940–947. 10.1016/j.leukres.2014.05.009 24939216

[B54] White E. (2012). Deconvoluting the context-dependent role for autophagy in cancer. *Nat. Rev. Cancer* 12 401–410. 10.1038/nrc3262 22534666 PMC3664381

[B55] Xie H. Xue Y. X. Liu L. B. Liu Y. H. Wang P. (2012). Role of RhoA/ROCK signaling in endothelial-monocyte-activating polypeptide II opening of the blood-tumor barrier: role of RhoA/ROCK signaling in EMAP II opening of the BTB. *J. Mol. Neurosci.* 46 666–676. 10.1007/s12031-011-9564-9569 21647708

[B56] Xu H. W. Huang Y. J. Xie Z. Y. Lin L. Guo Y. C. Zhuang Z. R. (2013). The expression of cytoglobin as a prognostic factor in gliomas: a retrospective analysis of 88 patients. *BMC Cancer* 13:247 10.1186/1471-2407-13-247 PMC3663650 23688241

[B57] Yao Y. Ma J. Xue Y. Wang P. Li Z. Li Z. (2015). MiR-449a exerts tumor-suppressive functions in human glioblastoma by targeting Myc-associated zinc-finger protein. *Mol. Oncol.* 9 640–656. 10.1016/j.molonc.2014.11.003 25487955 PMC5528701

[B58] Zhang H. Y. Zhang P. N. Sun H. (2009). Aberration of the PI3K/AKT/mTOR signaling in epithelial ovarian cancer and its implication in cisplatin-based chemotherapy. *Eur. J. Obstet. Gynecol. Reprod. Biol.* 146 81–86. 10.1016/j.ejogrb.2009.04.035 19540648

