# Supplementary material for: Autophagy Induction by Endothelial-Monocyte Activating Polypeptide II Contributes to the Inhibition of Malignant Biological Behaviors by the Combination of EMAP II with Rapamycin in Human Glioblastoma
Source: Front Mol Neurosci. 2015 Dec 1;8:74. doi: 10.3389/fnmol.2015.00074 (PMC4664732; doi:10.3389/fnmol.2015.00074)
Supplement: Supplementary file 1 [file Table_1.DOCX]

**Supplementary data**

Supplementary Table 1. Information of patient tissues.

All the patients provided written informed consent, and the study was approved by the Ethics Committee of Shengjing Hospital of China Medical University. The grade was identified according to the WHO classification by experienced clinical pathologists. Tumors used in this study were from newly diagnosed and treatment naive patient.

| Sample ID | Age | Sex | Histology type | WHO Grade | Distant metastasis | Predominant lobe of tumor location |
| --- | --- | --- | --- | --- | --- | --- |
| GBM-1` | 62 | F | GBM | Ⅳ | Without | Frontal |
| GBM-2 | 58 | F | GBM | Ⅳ | Without | Frontal |
| GBM-3 | 59 | M | GBM | Ⅳ | Without | Frontal |
| GBM-4 | 65 | M | GBM | Ⅳ | Without | Frontal |
| GBM-5 | 61 | M | GBM | Ⅳ | Without | Frontal |
